# FLT3L and granulocyte macrophage colony-stimulating factor enhance the anti-tumor and immune effects of an HPV16 E6/E7 vaccine

**DOI:** 10.18632/aging.102494

**Published:** 2019-12-24

**Authors:** Zhenzhen Ding, Hua Zhu, Laiming Mo, Xiangyun Li, Rui Xu, Tian Li, Liang Zhao, Yi Ren, Yunsheng Xu, Rongying Ou

**Affiliations:** 1 Department of Dermatovenereology, Yuyao People’s Hospital of Zhejiang Province, Yuyao, Zhejiang 315400, China; 2 Department of Dermatovenereology, The Seventh Affiliated Hospital, Sun Yat-sen University, Shenzhen, Guangdong 518107, China; 3 Department of Gynecology and Obstetrics, The First Affiliated Hospital of Wenzhou Medical University, Wenzhou, Zhejiang 325000, China; 4 Department of Clinical Laboratory, The Seventh Affiliated Hospital, Sun Yat-sen University, Shenzhen, Guangdong 518107, China; 5 Department of Gynecology and Obstetrics, The Seventh Affiliated Hospital, Sun Yat-sen University, Shenzhen, Guangdong 518107, China; 6 Laboratory for Advanced Interdisciplinary Research, Institutes of Translational Medicine, The First Affiliated Hospital of Wenzhou Medical University, Wenzhou, Zhejiang 325000, China; 7 Department of Biomedical Sciences, Florida State University College of Medicine, Tallahassee, FL 32304, USA

**Keywords:** HPV16 vaccine, FLT3L, GM-CSF

## Abstract

HPV16 infections promote the development and progression of cervical cancer. We investigated Fms-like Tyrosine Kinase 3 Ligand and granulocyte macrophage colony-stimulating factor as new adjuvants to an HPV16 vaccine. C57BL/6 mice were immunized by intramuscular injections of HPV16 E6/E7 plasmids every two weeks, three times in all. An *in vivo* imaging system was used to observe tumor growth and metastasis. Pathological changes to the heart, liver, spleen, lungs, brain and kidneys were recorded, and the survival rate of the mice was determined. The constructed HPV16 E6/E7 vaccine had no notable side effects in terms of physiological or biochemical indexes. Fms-like Tyrosine Kinase 3 Ligand and granulocyte macrophage colony-stimulating factor increased the inhibitory effects of the HPV16 E6/E7 vaccine on tumor growth and metastasis *in vivo.* The HPV16 E6/E7 vaccine enhanced the survival of mice and increased their serum-specific antibody and interferon-γ levels. Fms-like Tyrosine Kinase 3 Ligand and granulocyte macrophage colony-stimulating factor augmented these effects. In a cytotoxic lymphocyte killing test, Fms-like Tyrosine Kinase 3 Ligand and granulocyte macrophage colony-stimulating factor improved the ability of splenic lymphocytes from HPV16 E6/E7-vaccinated mice to kill B16 cells. As Fms-like Tyrosine Kinase 3 Ligand and granulocyte macrophage colony-stimulating factor enhanced the anti-tumor and immune effects of the HPV16 vaccine, these adjuvants should be considered for the treatment of cervical cancer.

## INTRODUCTION

Cervical cancer is the second most common type of malignant cancer in women worldwide. About 500,000 new cases occur each year, and nearly half of the cases result in death. Human papillomavirus (HPV), which mainly infects human skin or mucosal epithelial cells, can cause benign or malignant lesions at its infection sites [1]. High-risk HPV infections are strongly associated with cervical cancer [2], and about 50% of cervical cancer cases involve HPV16 infections [2, 3]. There are currently no effective clinical measures to prevent HPV16 infections or advanced cervical cancer recurrence. Therefore, the development of effective vaccines to prevent and treat HPV16 infections is very important for patients with or at risk for cervical cancer.

Since HPV is difficult to culture *in vitro*, HPV vaccine development has progressed slowly [4]. However, with the rapid development of DNA recombination techniques, eukaryotic recombinant expression vectors carrying antigen-encoding genes (e.g., nucleic acid vaccines) can be directly injected into the bodies of animals, causing a specific immune response [5]. Nucleic acid vaccines can prevent diseases by inducing humoral and cellular immunity [6], and thus offer a new way of treating infectious diseases such as cervical cancer [7]. Although nucleic acid vaccines have been quickly and extensively developed, their overall immune effects remain poor [8].

Previous research has demonstrated that the HPV16 E6 and E7 proteins (encoded by the viral early genes *E6* and *E7*) bind to the p53 and retinoblastoma proteins, respectively [9]. The E6 protein efficiently abrogated the growth arrest and apoptosis induced by p53, while the E7 protein promoted cell proliferation by degrading the tumor suppressor protein pRB (retinoblastoma) [10]. Continued E6 and E7 protein expression is necessary for the growth and tumorigenicity of cervical carcinoma cells [11]. Zhou et al. found that HPV16 E6/E7 promoted the progression of cervical cancer [12].

Granulocyte macrophage colony-stimulating factor (GM-CSF) has been reported to increase the anti-tumor effects of cancer vaccines [13]. In addition, Fms-like Tyrosine Kinase 3 Ligand (FLT3L) has been reported to accelerate dendritic cell maturation [14]. Zhang et al. found that the use of FLT3L as an adjuvant enhanced the protection of vaccinated mice against a lethal rabies virus challenge [15]. Adjuvant FLT3L also improved the anti-tumor effects of a DNA plasmid carrying Mucin 1 [16]. In the present study, we explored the effects of FLT3L and GM-CSF on the anti-tumor and immune activities of an HPV16 E6/E7 vaccine.

## RESULTS

### The HPV16 E6/E7 plasmids were successfully constructed

**Figure 1:**
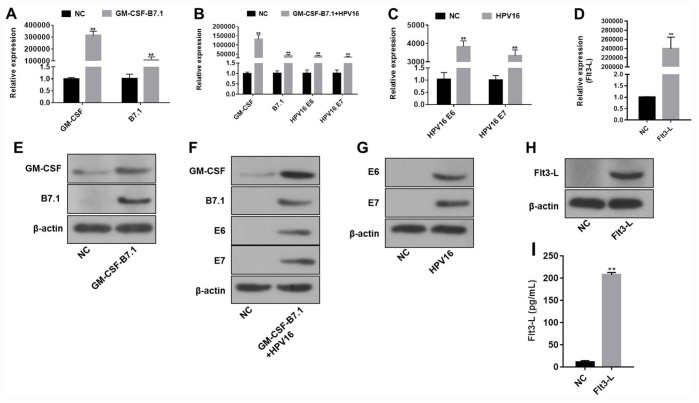
**The efficiency of the HPV16 E6/E7 plasmids was validated by qRT-PCR and Western blotting.** (**A**–**D**) The gene expression of pVAX1-IRES-GM-CSF-B7.1 (A), pVAX1-IRES-GM-CSF-B7.1-HPV16 E6/E7 (**B**), pIRES-neo3-HPV16 E6/E7 (**C**) and pVAX1-IRES-FLT3L (**D**) in B16 cells was detected by qRT-PCR. (**E**–**H**) The protein expression of pVAX1-IRES-GM-CSF-B7.1 (**E**), pVAX1-IRES-GM-CSF-B7.1-HPV16 E6/E7 (**F**), pIRES-neo3-HPV16 E6/E7 (**G**) and pVAX1-IRES-FLT3L (**H**) in B16 cells was detected by Western blotting. (**I**) FLT3L levels in B16 cells transfected with pVAX1-IRES-FLT3L were detected by ELISA. **P < 0.01 compared with the pVAX1-IRES or pIRES-neo3 group.

The pVAX1-IRES-GM-CSF-B7.1, pVAX1-IRES-FLT3L, pVAX1-IRES-GM-CSF-B7.1-HPV16 E6/E7 and pIRES-neo3-HPV16 E6/E7 plasmids were designed and constructed according to the gene reference sequences and vector sequences in the National Center for Biotechnology Information. As indicated in Supplementary Figure 1, the product sizes of the recombinant plasmids were as expected, and the sequences of the plasmids were correct. In addition, qRT-PCR, Western blotting and ELISA were used to detect the expression of the vector components. As shown in Figure 1, pVAX1-IRES-GM-CSF-B7.1 transfection significantly increased GM-CSF and B7.1 expression in B16 cells. Transfection with pVAX1-IRES-FLT3L significantly increased FLT3L levels in B16 cells and cell supernatants. Finally, transfection with pIRES-neo3-HPV16 E6/E7 or pVAX1-IRES-GM-CSF-B7.1-HPV16 E6/E7 significantly increased the expression of HPV16 E6/E7 in B16 cells. These data indicated that the fusion genes of the pVAX1-IRES-GM-CSF-B7.1, pVAX1-IRES-FLT3L, pVAX1-IRES-GM-CSF-B7.1-HPV16 E6/E7 and pIRES-neo3-HPV16 E6/E7 plasmids were constructed successfully.

### The nucleic acid vaccine was highly safe in mice

**Figure 2:**
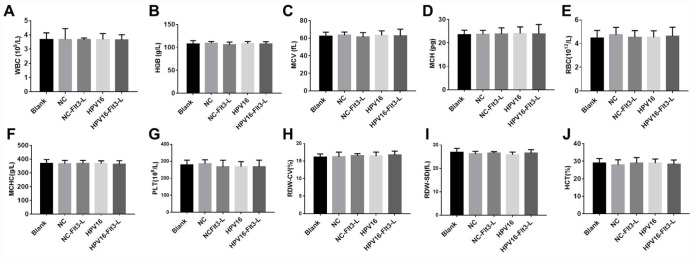
**The nucleic acid vaccine had no effect on blood counts in mice.** (**A**–**J**) The plasma levels of WBC (**A**), HGB (**B**), MCV (**C**), MCH (**D**), RBC (**E**), MCHC (**F**), PLT (**G**), RDW-CV (**H**), RDW-SD (**I**) and HCT (**J**) in mice were detected with a cellular counter.

**Figure 3:**
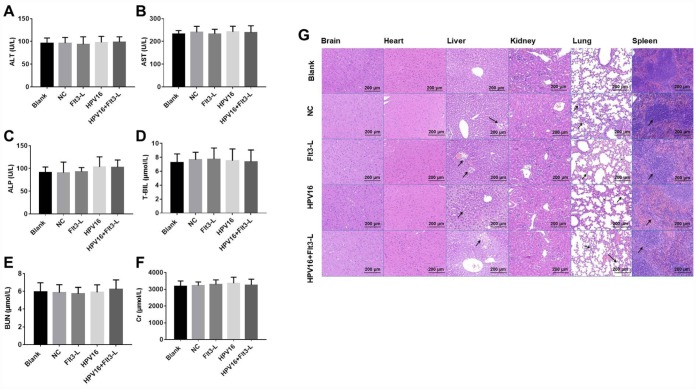
**The nucleic acid vaccine did not affect blood biochemical indexes and had a good safety profile in mice.** (**A**–**F**) The plasma levels of ALT (**A**), AST (**B**), ALP (**C**), T-BIL (**D**), BUN (**E**) and CR (**F**) in mice were detected with an automatic biochemical analyzer. After the mice were sacrificed, samples of their hearts, brains, kidneys, spleens, lungs and livers were immediately collected and fixed with a 10% formaldehyde solution. (**G**) Tissues of mice in the blank, pVAX1-IRES (NC), pVAX1-IRES-GM-CSF-B7.1-HPV16 E6/E7 (HPV16), pVAX1-IRES-FLT3L (FLT3L) and pVAX1-IRES-GM-CSF-B7.1-HPV16 E6/E7-FLT3L (HPV16+FLT3L) groups were then dehydrated, paraffin-embedded, sliced (4-μm thick), stained with hematoxylin and eosin and observed with an optical microscope. Black arrow indicates a few inflammatory vacuoles in liver tissues, slight thickening of alveolar wall, shrinkage of alveolar cavity, infiltration of inflammatory cells in lung tissues and shrinkage of lymphoid follicles in spleen tissues

In order to evaluate the safety of the HPV16 vaccine, we examined the blood physiology and biochemistry of the immunized mice. As indicated in Figures 2A–2J and 3A–3F, the HPV16 vaccine did not significantly alter the blood physiology and biochemical indexes of the mice. These results suggested that the constructed nucleic acid vaccine had no notable toxic side effects.

Next, we evaluated the effects of the vaccine on histopathological indexes of the immunized mice. As shown in Figure 3G, the HPV16 vaccine did not significantly impact histopathological indexes in the brain, heart and kidney tissues. However, we observed a few inflammatory vacuoles in the liver tissues; slight thickening of the alveolar wall, shrinkage of the alveolar cavity and infiltration of inflammatory cells in the lung tissues; and shrinkage of lymphoid follicles in the spleen tissues. Thus, the HPV16 E6/E7 vaccine had a good safety profile.

### *E6* and *E7* were upregulated after transfection in B16 cells

**Figure 4:**
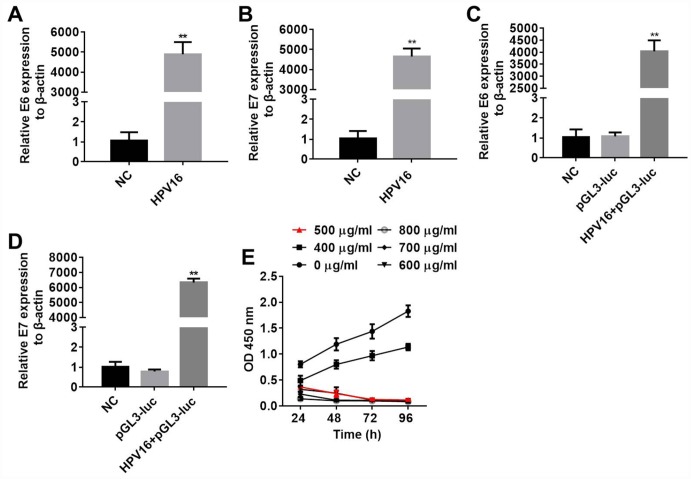
**B16 cells were stably transfected with pIRES-neo3 and pIRES-neo3-HPV16 E6/E7+pGL3-luc.** (**A**, **B**) *E6* and *E7* levels in B16 cells transfected with pIRES-neo3-HPV16 E6/E7 or pIRES-neo3 were verified by qRT-PCR. (**C**, **D**) *E6* and *E7* levels in B16 cells transfected with pIRES-neo3-HPV16 E6/E7-pGL3-luc were verified by qRT-PCR. (**E**) B16 cells were treated with G418 at concentrations of 0, 400, 500, 600, 700 and 800 μg/mL, and their proliferation was measured with an MTT assay at 24, 48, 72 and 96 h, respectively. ^**^P < 0.01 compared to the NC group.

Next, B16 cells were stably transfected with pIRES-neo3 or pIRES-neo3-HPV16 E6/E7 plasmids. In order to detect the efficacy of the transfections, we measured *E6* and *E7* expression in the B16 cells by qRT-PCR, and examined the proliferation of the cells through an MTT assay. As shown in Figure 4A–4D, *E6* and *E7* levels were significantly greater in cells stably transfected with pIRES-neo3-HPV16 E6/E7 than in cells stably transfected with pIRES-neo3. In addition, the optical density values of the pIRES-neo3 (NC), pGL3-luc (pGL3) and pIRES-neo3-HPV16 E6/E7+pGL3-luc (HPV16+pGL3) groups suggested that the stable strains were constructed successfully (Figure 4E). All these results indicated that B16 cells were stably transfected with pIRES-neo3 and pIRES-neo3-HPV16 E6/E7+pGL3-luc.

### The HPV16 E6/E7 protein was successfully purified

The transformed vector pGEX-4t-3-HPV16 E6/E7 was induced by isopropyl β-D-1-thiogalactopyranoside, and the HPV16 E6/E7 protein was purified as a source of antigens for subsequent animal experiments. As shown in Supplementary Figure 2, the molecular weight of the protein was about 170 kDa, in accordance with the theoretical size of the HPV16 E6/E7 protein. Given these results and the previous sequencing results, we concluded that the HPV16 E6/E7 protein was successfully purified. A BCA assay revealed that the concentration of HPV16 E6/E7 was 5.03 ± 0.14 mg/mL.

### FLT3L and GM-CSF enhanced the anti-tumor effects of the HPV16 E6/E7 vaccine *in vivo*

**Figure 5:**
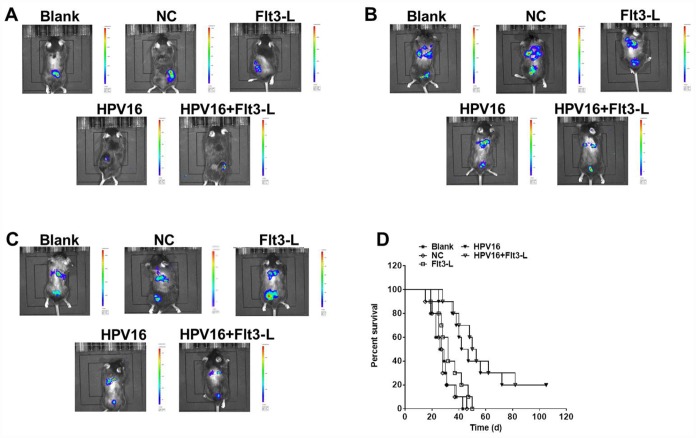
**The nucleic acid vaccine inhibited tumor metastasis *in vivo*.** Mice (6–8 weeks old) were randomly divided into five groups (five mice/group). The mice were immunized, and their tumor sizes and metastasis sites were detected with an IVIS after two weeks (**A**), four weeks (**B**) and six weeks (**C**). (**D**) The survival rates of mice in the blank, pVAX1-IRES (NC), pVAX1-IRES-FLT3L (FLT3L), pVAX1-IRES-GM-CSF-B7.1-HPV16 E6/E7 (HPV16) and pVAX1-IRES-GM-CSF-B7.1-HPV16 E6/E7-FLT3L (HPV16+FLT3L) groups were calculated at different time points.

**Figure 6:**
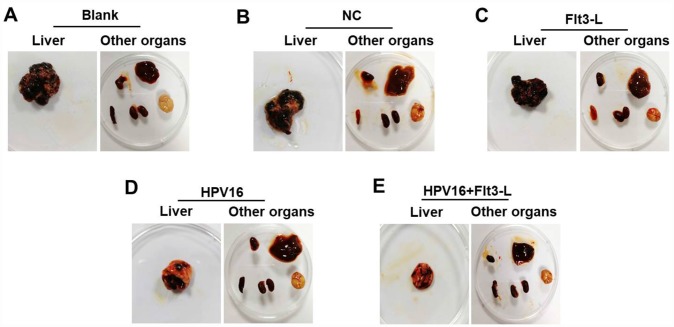
**The nucleic acid vaccine inhibited hepatic metastasis *in vivo*.** (**A**–**F**) Mice (n=25, five mice/group) in the blank, pVAX1-IRES, pVAX1-IRES-FLT3L, pVAX1-IRES-GM-CSF-B7.1-HPV16 E6/E7 and pVAX1-IRES-GM-CSF-B7.1-HPV16 E6/E7-FLT3L groups were injected with HPV16 E6/E7+Luc stable cells. After four weeks, they were sacrificed for the observation of tumor metastasis to other organs (heart, liver, spleen, lung, kidney and brain tissues).

We next investigated the anti-tumor effects of the HPV16 E6/E7 vaccine in mice subcutaneously inoculated with cells. As indicated in Figure 5A–5C, the HPV16 E6/E7 vaccine significantly inhibited tumor metastasis in these mice, and FLT3L significantly enhanced this inhibition. The HPV16 E6/E7 vaccine also markedly increased the survival rate of the mice, and FLT3L augmented this effect (Figure 5D). FLT3L notably enhanced the inhibitory effect of the HPV16 E6/E7 vaccine on hepatic metastasis; however, there was no significant difference in the morphology of the heart, spleen, kidney or brain between the groups (Figure 6A–6F). These results demonstrated that GM-CSF and FLT3L significantly enhanced the anti-tumor effects of the HPV16 E6/E7 vaccine.

### FLT3L and GM-CSF significantly enhanced the immune effects of the HPV16 E6/E7 vaccine

**Figure 7:**
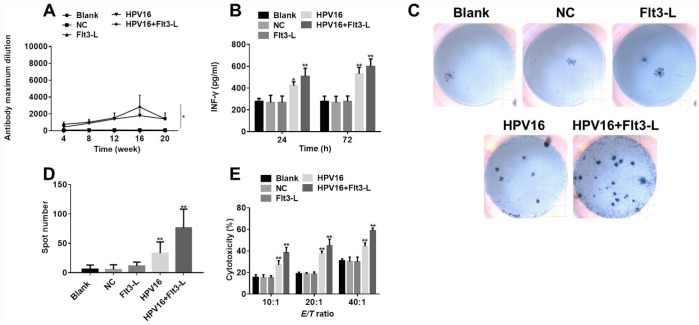
**The nucleic acid vaccine exhibited significant immune and tumor-killing effects.** (**A**) The titer of serum specific antibodies in mice was detected by ELISA at 4, 8, 12, 16 or 20 weeks. (**B**) IFN-γ levels in the culture supernatants of splenic lymph nodes co-cultured with stably transfected pIRES-neo3-HPV16 E6/E7 B16 cells were detected by ELISA. (**C**) The results of the ELISpot assay are pictured. (**D**) The ELISpot assay was used to detect the number of splenic lymphocytes secreting IFN-γ when the cells were co-cultured with stably transfected pIRES-neo3-HPV16 E6/E7 B16 cells. (**E**) The killing activities of splenic lymphocytes from pVAX1-IRES-GM-CSF-B7.1-HPV16 E6/E7 (HPV16) mice towards B16 cells stably transfected with HPV16 E6/E7 were tested with a cytotoxic lymphocyte killing test at lymphocyte:B16 cell ratios of 10:1, 20:1 and 40:1. ^*^P < 0.05, ^**^P < 0.01 compared to the NC group.

Next, to evaluate the immune effects of the HPV16 E6/E7 vaccine, we used ELISA and ELISpot to detect the serum levels of specific antibodies and the cytokine IFN-γ in the mice. As shown in Figure 7A, the HPV16 E6/E7 vaccine significantly increased the titer of specific antibodies in the serum, and FLT3L further increased the titer. Moreover, the level of IFN-γ was significantly increased by the HPV16 E6/E7 vaccine and further enhanced by FLT3L (Figure 7B). An ELISpot assay revealed that GM-CSF increased the number of spleen cells secreting IFN-γ, and FLT3L significantly enhanced this effect (Figure 7C, 7D). All these data indicated that GM-CSF and FLT3L enhanced the immune response to the HPV16 E6/E7 vaccine.

### FLT3L and GM-CSF enhanced the anti-tumor effects of the HPV16 E6/E7 vaccine *in vitro*

Finally, to confirm the anti-tumor effects of the HPV16 E6/E7 vaccine *in vitro*, we performed a cytotoxic lymphocyte killing test to determine the B16-cell-killing activity of splenic lymphocytes from the various groups of mice. As shown in Figure 7E, the killing activity of splenic lymphocytes was significantly greater in the HPV16 group than in the control group, indicating that GM-CSF significantly enhanced the tumor-killing effects of the HPV16 E6/E7 vaccine. FLT3L further increased these effects. All these data confirmed that GM-CSF and FLT3L promoted the anti-tumor effects of the HPV16 E6/E7 vaccine.

## DISCUSSION

Due to the tumorigenic characteristics of HPV, therapeutic vaccines with HPV16 transforming proteins E6 and E7 as target antigens could provide new methods of treating cervical cancer [18–21]. The inhibition of HPV16 E6 and E7 expression *in vitro* has been reported to suppress tumorigenesis [22, 23]. To further reduce the oncogenic potential of E6 and E7, we generated a nucleic acid vaccine in which their coding sequences were fused.

In this research, FLT3L and GM-CSF enhanced the anti-tumor effects of the HPV16 E6/E7 vaccine, suggesting that these adjuvants probably reduced the oncogenic effects of E6 and E7. Similarly, a previous report indicated that FLT3L could inhibit tumorigenesis [24]. Our results also indicated that the HPV16 E6/E7 vaccine had very limited side effects in mice. Demmerath et al. found that FLT3L inhibited the tumorigenesis of liver cancer without toxic effects [25]. These data suggested that FLT3L and GM-CSF increased the anti-tumor effects of the HPV16 E6/E7 vaccine without systemic toxicity *in vivo*.

The HPV genome also encodes two late proteins (L1 and L2). L1 and L2 are viral capsid proteins that can promote the anti-tumor effects of vaccines [26]. The effects of these two proteins on the HPV16 E6/E7 vaccine will be investigated in the future.

We also evaluated the T-cell-mediated immune response induced by immunization with the HPV16 E6/E7 vaccine. The HPV16 E6/E7 vaccine significantly increased IFN-γ cytokine levels, and adjuvant FLT3L further increased the immune effects of the vaccine. A previous study indicated that a GM-CSF expression vector was a key inducer of protective immune responses [27]. Our results confirmed these findings, as IFN-γ is regarded as a critical cytokine in T-cell responses [28]. Furthermore, Gill et al. found that adjuvant very small size proteoliposomes (VSSPs) increased the immune effects of an HPV16 vaccine by inducing T-cell responses [29], similar to the results of the current research. Our data indicated that FLT3L and GM-CSF enhanced the immune effects of the HPV16 E6/E7 vaccine by activating the T-cell-mediated immune response.

In conclusion, FLT3L and GM-CSF significantly enhanced the anti-tumor and immune effects of the HPV16 E6/E7 vaccine. Thus, the inclusion of these adjuvants could be considered in novel strategies for the treatment of cervical cancer.

## MATERIALS AND METHODS

### Cell culture and experimental animals

B16 cells were provided by Shanghai Fu Xiang Biotechnology Co., Ltd. Cell stocks were maintained in 5% CO_2_ at 37°C with Roswell Park Memorial Institute (RPMI) medium containing 10% fetal bovine serum, and the concentration was maintained at 2 × 10^5^ cells/mL.

C57BL/6 mice were purchased from the Laboratory Animal Center of Huazhong Agricultural University and housed within a dedicated specific pathogen-free facility with alternating 12-h periods of light and darkness at a constant temperature of 18–23°C and 55–65% humidity. All animal experiments were performed in accordance with institutional guidelines, following a protocol approved by the Ethics Committee of The Seventh Affiliated Hospital, Sun Yat-sen University.

### Vector construction

The All the plasmids in this study were designed and constructed according to the gene reference sequences and vector sequences in the National Center for Biotechnology Information. Then, enzyme digestion and sequencing were carried out.

### Quantitative real-time PCR

Total RNA was extracted with TRIzol® Reagent (Invitrogen, Carlsbad, CA, USA). Quantitative real-time polymerase chain reaction (qRT-PCR) experiments were performed with the TaqMan PCR Master Mix according to the manufacturer’s protocol. The 2^-ΔΔCT^ method was used to calculate the relative gene expression, and β-actin was used as an internal control. The incubation was initiated at 37°C for 15 min, followed by 95°C for 30 s and 60°C for 34 s for 40 cycles. The primers are listed in Table 1.

**Table 1:** Primer sequences.

**Gene**	**Forward primer**	**Reverse primer**
GM-CSF	GGCAGCCTCACCAAGCTCAAG	GCAGTCAAAGGGGATGACAAG
B7.1	GCTGGCTGGTCTTTCTCAC	ACTCGTATGTGCCCTCGTC
HPV16 E6	CAGGAGCGACCCAGAAAGT	AACGGTTTGTTGTATTGCTGTTC
HPV16 E7	AGGAGGATGAAATAGATGG	TTGTACGCACAACCGAAGC
FLT3L	AGCTGTCTGACTACCTGCTTCA	GATGTTGGTCTGGACGAAGC
β-actin	CACGATGGAGGGGCCGGACTCATC	TAAAGACCTCTATGCCAACACAGT

### Western blot

The protein concentrations were determined with a bicinchoninic acid (BCA) protein assay kit (Beyotime, Shanghai, China). Then, equal amounts of the protein extracts (30 μg) were electrophoretically separated through a 10% sodium dodecyl sulfate polyacrylamide gel. The proteins were transferred to polyvinylidene fluoride membranes (Thermo Fisher Scientific, Waltham, MA, USA) for 2 h and blocked with 4% skim milk powder in Tris-buffered saline-Tween for another 1 h. The membranes were incubated with the following primary antibodies overnight at 4°C: GM-CSF antibody (1:1000; Affinity, Cambridge, UK), B7.1 antibody (1:1000; Affinity), HPV16 E7 antibody (1:300; Santa Cruz Biotechnology, Dallas, TX, USA), FLT3L antibody (1:1000; Abcam, Cambridge, MA, USA), HPV18 E7 (1:1000; Abcam), luciferase antibody (1:1000; Abcam) and β-actin antibody (1:1000; Wuhan Doctorate Bioengineering, Wuhan, China). After being washed with phosphate-buffered saline, the membranes were then incubated with secondary antibody (1:5000; Abcam) at room temperature for 1 h. Finally, the immunoreactivity was detected with the enhanced chemiluminescence reagent (Santa Cruz Biotechnology).

### Stable transfection

B16 cells were stably transfected with pIRES-neo3 or pIRES-neo3-HPV16 E6/E7, and the cells were screened by exposure to G418 (0, 400, 500, 600, 700 and 800 μg/mL). Then, cell proliferation was detected with an MTT assay, and the efficiency of transfection was verified by qRT-PCR.

### Evaluation of vaccine safety in mice

C57BL/6 mice (6–8 weeks old) were randomly divided into five groups (five mice/group). The mice were immunized through the intramuscular injection of plasmids once every two weeks for a total of three times. The mice in blank group were injected with normal saline. The mice in the negative control (NC) group were injected with the pVAX1-IRES control plasmid. The mice in the FLT3L group were injected with pVAX1-IRES-FLT3L. The mice in the HPV16 group were injected with pVAX1-IRES-GM-CSF-B7.1-HPV16 E6/E7. The mice in the HPV16+FLT3L group were injected with pVAX1-IRES-GM-CSF-B7.1-HPV16 E6/E7-FLT3L. Each plasmid was prepared in sterilized saline. The dose was 50 μg/mouse for the HPV16 vaccine and 50 μg/mouse for the adjuvant, and the injection volume was 100 μL.

Ten days after the last injection, plasma was obtained from the mice. The plasma levels of Red Blood Cell (RBC), hemoglobin (HB), Red blood cell specific volume (HCT), erythrocyte mean corpuscular volume (MCV), erythrocyte mean corpuscular hemoglobin (MCH), erythrocyte mean corpuscular hemoglobin concentrate (MCHC), blood platelet (PLT), white blood cell (WBC), red blood cell distribution width-coefficient of variation (RDW-CV) and red blood cell distribution width- stable disease (RDW-SD) were detected with a cellular counter (Beckman, Fullerton, CA, USA), while the plasma levels of ALT, AST, ALP, T-BIL, BUN, Cr, TP, ALB and GLU were measured with an automatic biochemical analyzer (Rayto, Shenzhen, China). Then, the mice were sacrificed, and their hearts, brains, kidneys, spleens, lungs and livers were immediately removed and fixed with a 10% formaldehyde solution. The tissues were then dehydrated, paraffin-embedded and sliced (4-μm thick). Hematoxylin and eosin staining was performed so that histopathological changes could be observed under an optical microscope.

### Immunization and *in* *vivo* imaging system (IVIS) investigation

An additional five groups of mice (five mice per group) treated in the same manner as those described above were used for tumorigenesis experiments. After being immunized, the mice were subcutaneously inoculated with 2×10^6^ (0.1 mL) HPV16 E6/E7 cells (luciferase). After 8 weeks of inoculation, the mice were anesthetized by an intraperitoneal injection of 3% sodium pentobarbital and were observed with an IVIS (Perkin Elmer, Waltham, MA, USA). The survival of the mice was observed for 15 weeks. At the end of the study, the hearts, livers, spleens, lungs, kidneys and brain tissues of the mice were collected so that tumor metastasis could be observed.

### Cytotoxic lymphocyte killing test

The spleens of the mice in each group were ground with a stainless-steel wire mesh. Each resulting single-cell suspension was slowly loaded into a lymphocyte-separating solution. After centrifugation at 2000 × *g* for 10 min, the lymphocytes were collected. The lymphocytes were washed twice with RPMI 1640 medium and co-cultured with B16 cells (stably transfected with HPV16 E6/E7 and pretreated with 50 μg/mL mitomycin C for 1 h) for five days at 10:1, 20:1 or 40:1 ratios in medium containing 20 U/mL recombinant mouse interleukin-2 and 10 μg/mL concanavalin A. The killing activity of the cytotoxic lymphocytes towards the target cells was calculated by the following formula.

$$\begin{array}{ccc} Percent\;cytotoxicity & = & \frac{\begin{array}{c} Experimental\;value–Effector\;spontaneous\;value\;\\ –\;Target\;spontaneous\;value\times xperi \end{array}}{Target\;maximum\;value–Target\;spontaneous\;value} \end{array}$$

### Detection of antigen-specific immune responses

Mouse serum was collected by tail-cutting on the 4^th^, 8^th^, 12^th^, 16^th^ and 20^th^ weeks after the first immunization for the determination of antigen-specific immune responses. A recombinant HPV16 E6/E7 antibody was applied to a 96-well plate at 37°C for 2 h, and then was sealed with 10% calf serum for 1 h. Different dilutions of the serum samples were then added to the wells and allowed to react for 1 h. Finally, excess horseradish peroxidase-labeled goat anti-mouse antibodies were added and incubated for 1 h. Color developers A and B were added to the dark box and observed every 10 min until the negative control (blank serum) was slightly better. Finally, 2M sulfuric acid was added to stop the reaction, and the optical density was measured at 492 nm.

### Preparation of spleen lymph node cells and induction of interferon (IFN)-γ cytokine secretion

Three weeks after the last immunization, the mice were sacrificed and their splenic lymphocytes were collected. The lymphocytes were co-cultured with B16 (HPV16 E6/E7) cells for 24 or 72 h. Then, the cell supernatants were collected, and IFN-γ levels were detected with an enzyme-linked immunosorbent assay (ELISA) kit (Excellbio, Shanghai, China) according to the manufacturer’s instructions.

### Splenic lymphocyte detection

Three weeks after the last immunization, the mice were sacrificed and their splenic lymphocytes were collected. Then, the lymphocytes were co-cultured with B16 (HPV16 E6/E7) cells for 72 h. The number of splenic lymphocytes secreting IFN-γ was detected with an enzyme-linked immunospot (ELISpot) assay [17] according to the manufacturer’s instructions.

### Statistical analysis

Each experiment was performed at least three independent times. All values are expressed as the mean ± standard deviation. Comparisons among multiple groups were made with one-way analysis of variance followed by Tukey’s test (GraphPad Prism7). For all tests, *P* values <0.05 were considered statistically significant.

## Supplementary Material

Supplementary Figures
