# Supplementary material for: FLT3L and granulocyte macrophage colony-stimulating factor enhance the anti-tumor and immune effects of an HPV16 E6/E7 vaccine
Source: Aging (Albany NY). 2019 Dec 24;11(24):11893–904. doi: 10.18632/aging.102494 (PMC6949056; doi:10.18632/aging.102494)
Supplement: Supplementary Figures [file aging-11-102494-s001..pdf]

SUPPLEMENTARY FIGURES

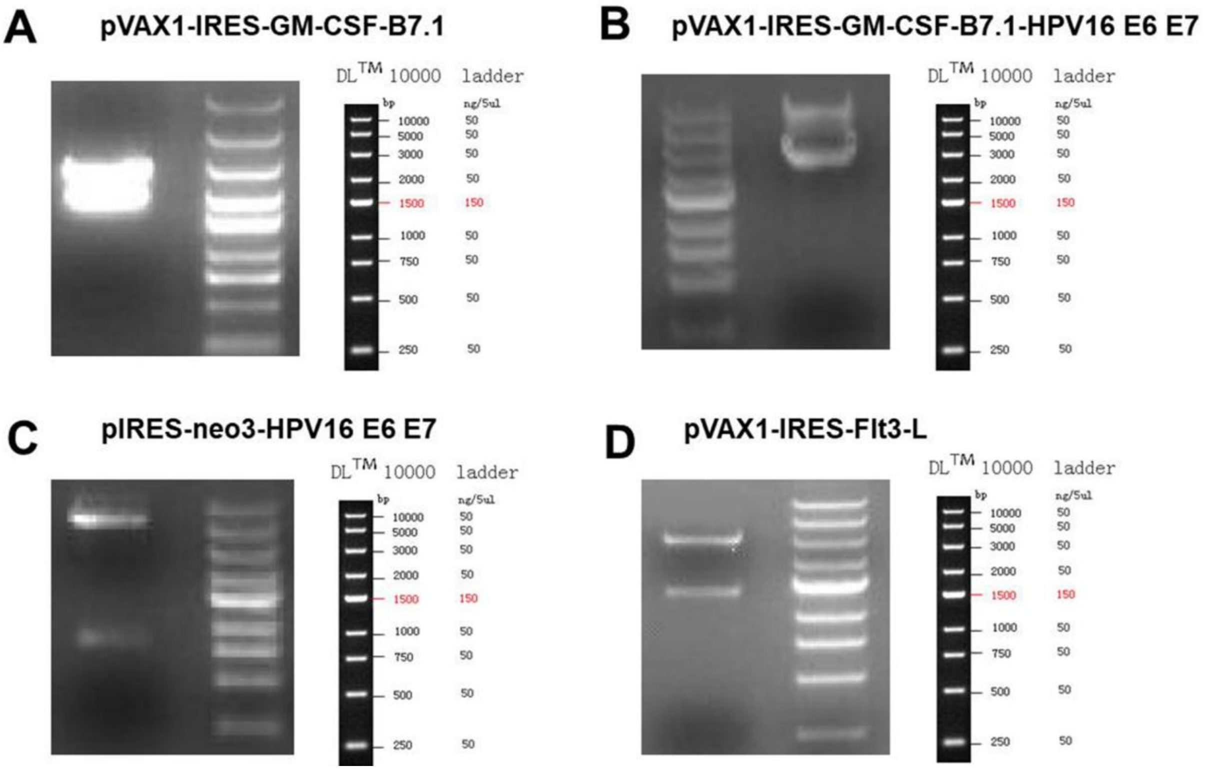

Supplementary Figure 1. The HPV16 E6/E7 plasmids were constructed successfully. Validation of the pVAX1-IRES-GM-CSF-B7.1 (**A**), pVAX1-IRES-GM-CSF-B7.1-HPV16 E6/E7 (**B**), pIRES-neo3-HPV16 E6/E7 (**C**) and pVAX1-IRES-FLT3L (**D**) vaccines.

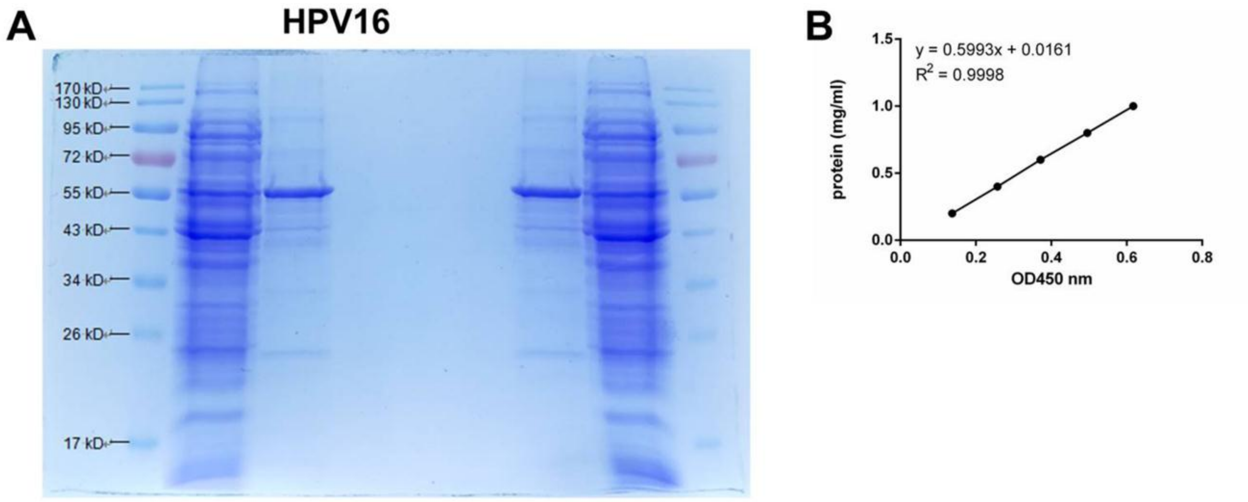

Supplementary Figure 2. The HPV16 E6/E7 protein was successfully purified. (**A**) The gel map of the sodium dodecyl sulfate polyacrylamide gel is shown before and after protein purification. (**B**) A BCA assay was used to generate the standard curve of the purified protein.
